# Familiarization with ambulatory sleep and blood pressure monitoring is necessary for representative data collection

**DOI:** 10.14814/phy2.15843

**Published:** 2023-10-20

**Authors:** Kasey Kleiber, Caroline J. Smith, Steven D. Beck, Adam Hege, Makenzie Corgan, Crystal A. West, Lainey Hunnicutt, Scott R. Collier

**Affiliations:** ^1^ Department of Public Health and Exercise Science Appalachian State University Boone North Carolina USA

**Keywords:** blood pressure, non‐rapid eye movement, rapid eye movement, sleep

## Abstract

Ambulatory sleep and blood pressure monitoring are gaining popularity as these can be completed in an individual's home. Little is known regarding the reliability of data and the time it takes to acclimate to the equipment. This study aimed to determine how many nights of wearing the monitoring equipment were required to restore sleep architecture and blood pressure data to baseline. It was hypothesized familiarization would be demonstrated by night 3. Ten male and 10 female subjects completed three nights of sleep and blood pressure recordings. At visit 1, the subjects were familiarized with the equipment and instructed to wear the Sleep Profiler{trade mark, serif} and SunTech Medical Oscar2 ambulatory blood pressure cuff simultaneously for three consecutive nights, then subjects returned the equipment. The percent of time spent in rapid eye‐movement (REM) sleep was statistically different on night 3 when compared to night 1. Wake‐after‐sleep onset and sleep latency were not statistically different between nights 1, 2, and 3. Systolic, diastolic, and pulse pressure were all significantly lower on night 3 compared to night 1. Cortical and autonomic arousals were statistically different on night 3. Ambulatory sleep and blood pressure monitoring need at least 3 nights for familiarization. The percent of time spent in REM sleep was statistically different on night 3 when compared to night 1. Systolic blood pressure, diastolic blood pressure, and pulse pressure were all significantly lower on night 3 compared to night 1. Cortical and autonomic arousals were statistically different on nights 3 and 2, respectively compared to night 1. Based on these findings, ambulatory sleep and blood pressure monitoring takes three nights before the data are reliable and the person is familiarized with the mode of measurement. Therefore, it is recommended to use at least three nights of data collection when using the Sleep Profiler and Oscar2 ambulatory blood pressure cuff in order for results to be valid and reliable.

## 1 INTRODUCTION

Sleep is an imperative, mandatory process that has been shown by both human and rodent studies to have implications in psychological and physiological health (Belenky et al., 2003; Rechtschaffen et al., 1983). PSG utilizes electroencephalogram (EEG) signals, electrooculogram (EOG) signals, electromyography (EMG) signals, and electrocardiogram (ECG) signals, among other parameters such as pulse oximetry and respiration (American Association of Sleep Technologists, 2018). EEG measures brain activity, EOG monitors eye activity, EMG monitors muscle activity, and ECG monitors heart activity. When combined, PSG gives a full picture of what is happening at a physiological level during sleep across the body. A less invasive and more economical option to PSG are portable sleep monitors that collect data in a home setting. The Sleep Profiler™ a commercially available sleep monitor, allows for more accurate data collection as it gathers EEG data to determine sleep architecture (Finan et al., 2016).

One important physiological change that occurs during sleep is the reduction of blood pressure (BP). This decline in pressures is known as nocturnal BP dipping and is classified by a 10%–20% decrease (Yano & Kario, 2012). The mechanism behind nocturnal BP dipping involves baroreflex sensitivity and resetting to a lower set point in conjunction with reductions in sympathetic nervous system activity (Conway et al., 1983; Sayk et al., 2007; Sherwood et al., 2002). The absence of nocturnal dipping, or less than a 10% reduction in pressure, is coined “non‐dipping” (Dolan et al., 2005; Fagard et al., 2008; Smolensky et al., 2007). Measuring nocturnal BP has become an important part in the monitoring and treatment of chronic diseases. Nocturnal BP can be used as a predictor of cardiovascular (CV) events (Cappuccio, 2020). When compared to daytime BP measurements, nocturnal BP recordings were more accurate in predicting adverse events including, but not limited to stroke, myocardial infarction (MI), and organ damage (Cappuccio, 2020). Sleep can affect BP measurements, with frequent arousals, fragmented sleep, and more time spent in stage N1 (light non‐REM sleep) resulting in blunted BP dipping (Mansoor, 2002; Matthews et al., 2008; Ross et al., 2014; Sherwood et al., 2002). Sleep continuity in conjunction with a greater amount of time spent in deep, restorative sleep is needed to mitigate non‐dipping status.

The use of nocturnal ambulatory BP recording is also considered to be an important tool in diagnosing and monitoring hypertension (Carey & Marwick, 2020). This method targets individuals with masked or white‐coat hypertension (Carey & Marwick, 2020). Since readings are taken throughout the day, outside of a clinical office, measurements are more accurate in diagnosing those with higher blood pressure (Carey & Marwick, 2020). Rather than dipping overnight, some individuals have rising nocturnal BP. Kario et al. (2020) showed a significantly higher risk of CVD and heart failure associated with a rising nocturnal BP pattern. This study concluded nocturnal BP patterns contribute more to chronic disease risk than daytime BP readings (Kario et al., 2020).

Since many sleep studies report on architecture variables within the first one to two nights of sleep, the purpose of this study is to determine how many nights of wearing the Sleep Profiler™ paired with an ambulatory BP monitor are required to restore BP and sleep architecture data to baseline in normotensive, college‐aged students. We hypothesize that the third night of continuous ambulatory sleep and BP measurement would be different from first night and more representative of sleep architecture for each subject.

## 2 METHODS

### 2.1 Subjects

Twenty normotensive (<130 mmHg systolic blood pressure [SBP] and <80 mmHg diastolic blood pressure [DBP]) male (*n* = 10) and female (*n* = 10) subjects between the ages of 18 and 25 years old were recruited at Appalachian State University for this study. Subjects were excluded from the study if their resting BP at visit 1 was above 130/80 mmHg or if they could not wear the equipment all three nights.

### 2.2 Procedure

Twenty subjects completed both sleep architecture and BP measurements on all three nights of data collection. The first visit consisted of anthropometric and resting BP measurements. Prior to visit 1, subjects were advised to refrain from eating and drinking caffeinated beverages at least 2 h prior to reporting to the laboratory. After providing their written informed consent, height and weight were measured without shoes, and the subject was instructed to rest seated for 5 min. After 5 min, BP was measured, the subject was given a 1‐min rest, and a subsequent measure was taken. If the measurements were not congruent, a third BP measurement was ascertained after a 1‐min rest and averaged. BP measurements were completed using an automated BP detection system (GE DinaMap, Pro 400v2, USA). Following BP measurements, the subject was familiarized with the Sleep Profiler™ (Advanced Brain Monitoring, Inc.) and the SunTech Medical Oscar2 (SunTech Medical, Inc.) ambulatory BP device prior to departing the laboratory. The Oscar2 was programmed to take and record BP measurements every 40 min during sleep. Subjects were instructed to wear both devices simultaneously for three consecutive nights, to sleep in their normal sleeping environment, and to avoid alcohol and caffeine late in the afternoon. At visit 2, subjects returned the equipment. Data were stored within both devices and downloaded to a laboratory computer following night 3 of data collection.

### 2.3 Treatment of the data

All data were analyzed for outliers, and descriptive statistics were determined for each category (SPSS, v.24, Chicago, IL, USA). Sleep and BP data were analyzed using a repeated measures ANOVA (group × time) to determine any differences in the outcome variables over successive nights. If significance was observed, a Bonferroni correction factor was used to determine where any differences were detected between nights. Significance was set at *p* ≤ 0.05, and all data are reported as mean ± standard deviation (SD).

## 3 RESULTS

**FIGURE 1:**
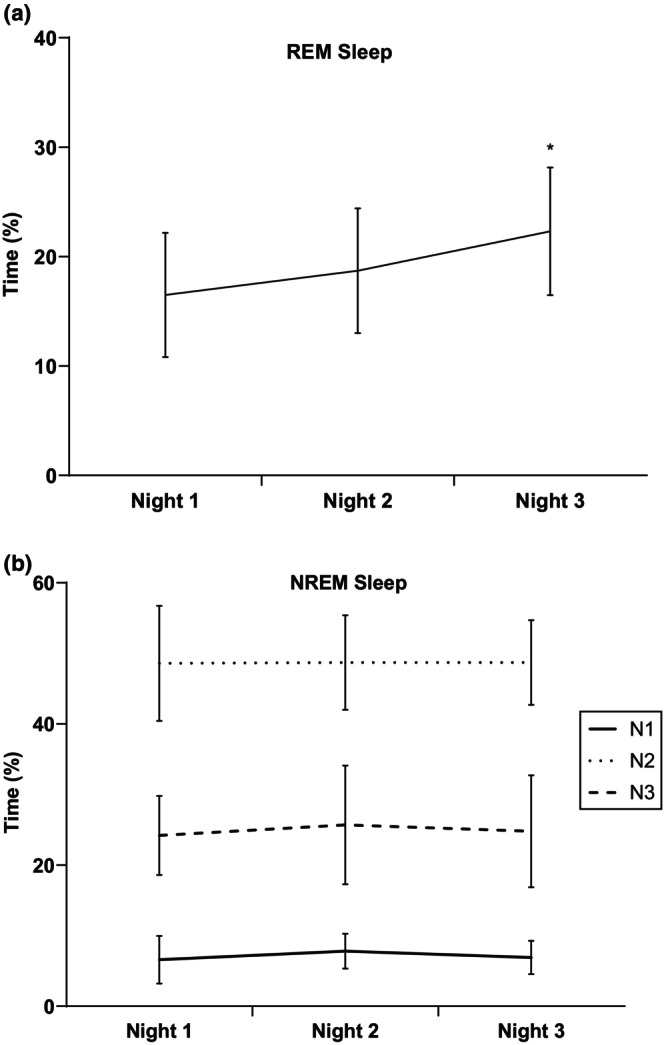
Percent of time spent in (a) rapid eye‐movement (REM) and (b) non‐rapid eye‐movement (NREM) sleep for nights 1, 2, and 3. *p < 0.05 versus night 1.

Descriptive characteristics of subjects are reported in Table 1. As shown in Figure 1a, there was a significant increase in the percentage of time spent in REM sleep on night 3 compared to night 1. There were no differences detected in the percentage of time spent in N1, N2, and N3 sleep across the three measured nights (Figure 1b).

**TABLE 1:** Descriptive characteristics of subjects.

Age (years)	Height (cm)	Weight (kg)	Resting SBP (mmHg)	Resting DPB (mmHg)
21.6 ± 2.2	163.9 ± 5.6	71.2 ± 8.2	119 ± 4.3	70 ± 2.8

*Note*: All data are reported as mean ± SD.

Abbreviations: DPB, diastolic blood pressure; SBP, systolic blood pressure.

**FIGURE 2:**
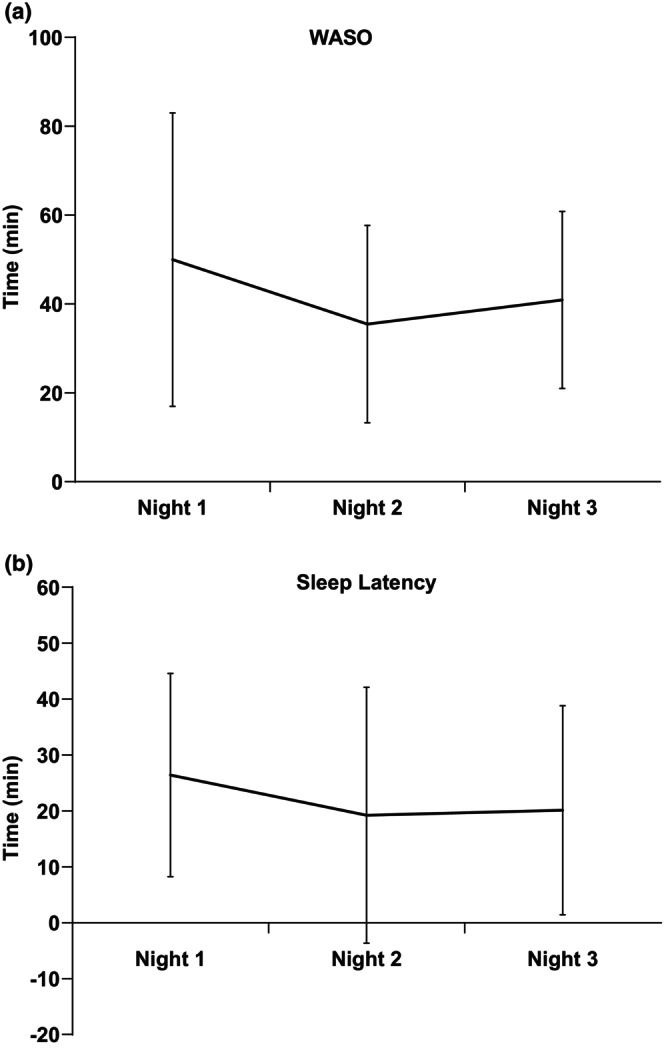
Time in minutes for (a) wake‐after‐sleep onset (WASO) and (b) sleep latency for nights 1, 2, and 3.

Wake‐after‐sleep onset (WASO) and sleep latency for nights 1, 2, and 3 were not statistically different (Figure 2). Although data are approaching significance toward a decrease in both WASO and sleep latency on nights 2 and 3.

**FIGURE 3:**
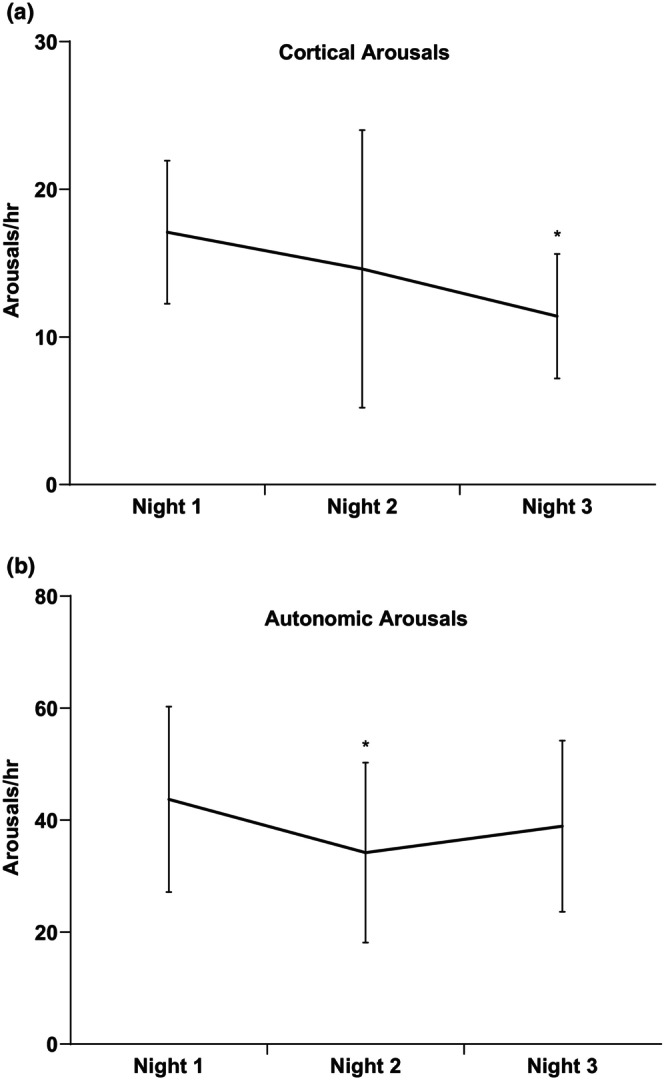
(a) Cortical and (b) autonomic arousals per hour for nights 1, 2, and 3. *p < 0.05 versus night 1. *p < 0.05 versus night 1.

Autonomic arousals were significantly lower on night 2 compared to night 1, but no differences were detected on night 3 (Figure 3). Cortical arousals were significantly lower on night 3 compared to night 1, but no differences were detected on night 2 (Figure 3).

**FIGURE 4:**
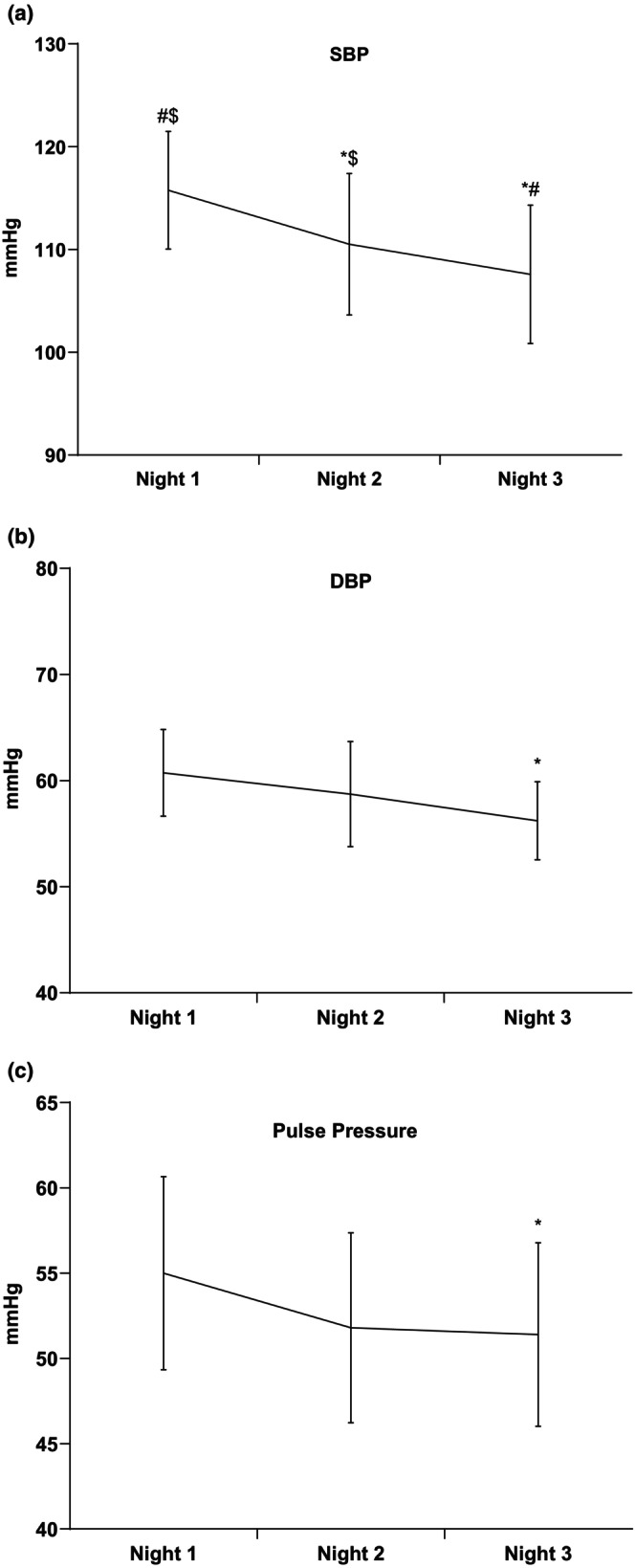
(a) Mean systolic blood pressure (SBP), (b) mean diastolic blood pressure (DBP), and (c) mean pulse pressure (PP) for nights 1, 2, and 3. Night 3 exhibited a lower mean DBP compared to night 1. *p < 0.05 versus night 1, ^#^ *p* < 0.05 versus night 2, ^$^ *p* < 0.05 versus night 3.

As shown in Figure 4a, mean systolic blood pressure (SBP) was statistically different between all nights. Mean diastolic blood pressure (DBP) was significantly lower on night 3 compared to night 1, but no differences were detected between nights 1 and 2 (Figure 4b).

## 4 DISCUSSION

To our knowledge, this is the first study to report sleep architecture combined with ambulatory BP monitoring within the subjects' own bed. The main finding of this study was that most sleep architecture and blood pressure variables showed an increase or decrease that is indicative of better sleep and reduced blood pressure on the second night with significance found with most variables on the third night of data collection.

N3 is the reparative and restorative stage that is essential for proper functioning. Typically, stage N3 comprises 20%–25% of a sleep episode (Spriggs, 2014). The values for percent of time spent in stage N3 fell within this range indicating that N3 sleep was not altered by cortical or autonomic arousals. Sayk et al. (2010) have shown that decreased time spent in N3 sleep exhibits deleterious effects on cardiovascular regulation during nocturnal dipping. This is evidenced when individuals were shifted via sleep interventional response, from N3 to N1 or N2, the nocturnal dip was attenuated, and sleep quality was decreased (Krystal & Edinger, 2008; Sayk et al., 2010).

Narrowing of the pulse pressure and lowering of the SBP and DBP from night 1 to night 3 indicate acclimatization to the sleep/pressure monitoring. We may be able to explain the changes in blood pressure between nights by the stress response from the subjects. Increased levels of cortisol, from the perceived stress of wearing the equipment for the first time, may contribute to elevated blood pressure on night 1 (Holt‐Lunstad & Steffen, 2007). Anticipation of the unknown has also been shown to increase the stress response leading to a worsened sleep quality, as Mohammad et al. (2019) have recently demonstrated. Our subjects showed the greatest amount of both cortical and autonomic arousals on night 1. Arousals made by the autonomic nervous system that is made from a sympathetic activation that was respiratory related or caused by leg movement would be an autonomic arousal (Krieger et al., 2003), while a cortical arousal is caused a spontaneous burst of EEG activity that lasts for more than three seconds (Amatoury et al., 2016) Both autonomic and cortical arousals contribute to sleep disturbances.

Rapid eye‐movement sleep continued to increase and was highest on night 3. An increase in REM sleep is indicative of improved sleep and has many physiological and psychological benefits, while decreases in REM sleep have been shown to decrease brain cell generation (Summer & Singh, 2023). Benefits of increase in REM include emotional processing, brain development, and memory consolidation (Summer & Singh, 2023). The increase in REM over the successive nights of measurement may be attributed to greater cortical integration (Clawson et al., 2018). Further, Ferreira et al. (2017) have shown that decreases in the prior night's REM sleep led to increases in melanin‐concentrating hormone (MCH) which has been shown to directly increase REM sleep. This data revealed the third night of data collection was the most accurate depiction of the subjects' sleep and blood pressure.

Monitoring sleep architecture and nocturnal BP gives important insight into an individual's overall health. Introducing new factors into a sleep environment will alter sleep architecture and BP measurements for the first two nights but will return to more normal sleep architecture on the third night. Therefore, three nights of data collection give an accurate representation of an individual's normal nocturnal physiological patterns, and the third night of data should be used for assessment.

## AUTHOR CONTRIBUTIONS

Kasey Kleiber: Conceived and designed research, performed experiments, analyzed data, interpreted results of experiments, and drafted manuscript. Caroline J. Smith: Analyzed data, interpreted results of experiments, and edited and revised manuscript. Steven D. Beck: Conceived and designed research, edited and revised manuscript. Adam Hege: Interpreted results of experiments, edited and revised manuscript. Makenzie Corgan: Edited and revised manuscript, approved final version of manuscript. Crystal A. West: Performed experiments, edited and revised manuscripts. Lainey Hunnicutt: Performed experiments, prepared figures, edited and revised manuscript, approved final version of manuscript. Scott R. Collier: Conceived and designed research, performed experiments, analyzed data, interpreted results of experiments, and approved final version of manuscript.

## ETHICS STATEMENT

The study was approved by the institutional review board and in accordance with the Declaration of Helsinki. All subjects were given and signed an informed consent.

## FUNDING INFORMATION

No funding information provided.
